# (2*E*)-1-(4-Bromo­phen­yl)-3-(4-fluoro­phen­yl)prop-2-en-1-one

**DOI:** 10.1107/S1600536810015485

**Published:** 2010-04-30

**Authors:** Grzegorz Dutkiewicz, K. Veena, B. Narayana, H. S. Yathirajan, Maciej Kubicki

**Affiliations:** a Department of Chemistry, Adam Mickiewicz University, Grunwaldzka 6, 60-780 Poznań, Poland; b Department of Studies in Chemistry, Mangalore University, Mangalagangotri 574 199, India; c Department of Studies in Chemistry, University of Mysore, Manasagangotri, Mysore 570 006, India

## Abstract

The title compound, C_15_H_10_BrFO, is isostructural with (2*E*)-1-(4-chloro­phen­yl)-3-(4-fluoro­phen­yl)prop-2-en-1-one [Qiu *et al.* (2006 ▶). *Acta Cryst.* E**62**, o3525–o3526], but the structures of other dihalogen analogues, without fluorine, are different, although they are also isostructural within the series. The mol­ecule is approximately flat, the dihedral angle between the ring planes being 8.49 (13)°. In the crystal structure, inter­molecular C—H⋯O, C—H⋯F and C—H⋯Br hydrogen bonds link mol­ecules into V-shaped ribbons running parallel to [101] and stacked with an inter­planar distance of approximately 3.53 Å (centroid–vcentroid distance = 3.857 Å)..

## Related literature

For general background to chalcones, see: Dhar (1981 ▶); Goto *et al.* (1991 ▶); Uchida *et al.* (1998 ▶); Indira *et al.* (2002 ▶); Sarojini *et al.* (2006 ▶). For a description of the Cambridge Structural Database, see: Allen (2002 ▶). For the isostructurality index, see: Kálmán *et al.* (1991 ▶). For related halogen derivatives, see: Ng, Razak, *et al.* (2006 ▶); Ng, Shettigar *et al.* (2006 ▶); Qiu *et al.* (2006 ▶); Wang *et al.* (2005 ▶); Yang *et al.* (2006 ▶).
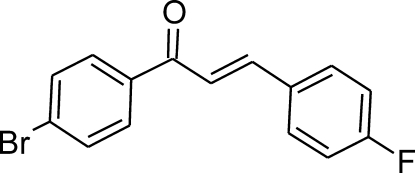


## Experimental

### 

#### Crystal data

C_15_H_10_BrFO
*M* *_r_* = 305.14
Monoclinic, 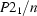

*a* = 4.0060 (5) Å
*b* = 23.1253 (12) Å
*c* = 13.4933 (9) Å
β = 96.344 (6)°
*V* = 1242.36 (19) Å^3^
*Z* = 4
Cu *K*α radiation
μ = 4.49 mm^−1^
*T* = 295 K
0.4 × 0.2 × 0.1 mm

#### Data collection

Oxford Diffraction SuperNova (single source at offset) Atlas diffractometer
Absorption correction: multi-scan (*CrysAlis PRO*; Oxford Diffraction, 2009 ▶) *T*_min_ = 0.386, *T*_max_ = 1.000
4548 measured reflections
2435 independent reflections
2299 reflections with *I* > 2σ(*I*)
*R*_int_ = 0.018

#### Refinement

*R*[*F* ^2^ > 2σ(*F* ^2^)] = 0.043
*wR*(*F* ^2^) = 0.116
*S* = 1.12
2435 reflections
203 parameters
All H-atom parameters refined
Δρ_max_ = 0.73 e Å^−3^
Δρ_min_ = −0.50 e Å^−3^

### 

Data collection: *CrysAlis PRO* (Oxford Diffraction, 2009 ▶); cell refinement: *CrysAlis PRO*; data reduction: *CrysAlis PRO*; program(s) used to solve structure: *SIR92* (Altomare *et al.*, 1993 ▶); program(s) used to refine structure: *SHELXL97* (Sheldrick, 2008 ▶); molecular graphics: *XP* (Siemens, 1989 ▶); software used to prepare material for publication: *SHELXL97*.

## Supplementary Material

Crystal structure: contains datablocks I, global. DOI: 10.1107/S1600536810015485/rz2439sup1.cif

Structure factors: contains datablocks I. DOI: 10.1107/S1600536810015485/rz2439Isup2.hkl

Additional supplementary materials:  crystallographic information; 3D view; checkCIF report

## Figures and Tables

**Table 1:** Hydrogen-bond geometry (Å, °)

*D*—H⋯*A*	*D*—H	H⋯*A*	*D*⋯*A*	*D*—H⋯*A*
C7—H7⋯O9^i^	0.98 (3)	2.62 (3)	3.512 (3)	151 (2)
C3—H3⋯O9^i^	0.99 (3)	2.41 (3)	3.358 (3)	160 (2)
C15—H15⋯Br1^ii^	1.02 (3)	2.92 (3)	3.845 (2)	151 (2)
C12—H12⋯F1^iii^	1.00 (3)	2.55 (3)	3.351 (3)	137 (2)

Symmetry codes: (i) 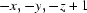; (ii) 
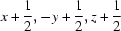
; (iii) 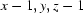.

**Fig. 1.**
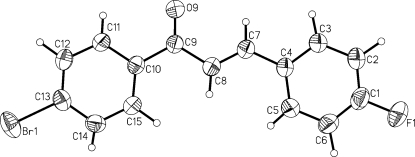
Perspective view of the title compound with labelling scheme and displacement ellipsoids drawn at 50% probability level. Hydrogen atoms are depicted as spheres with arbitrary radii.

**Fig. 2.**
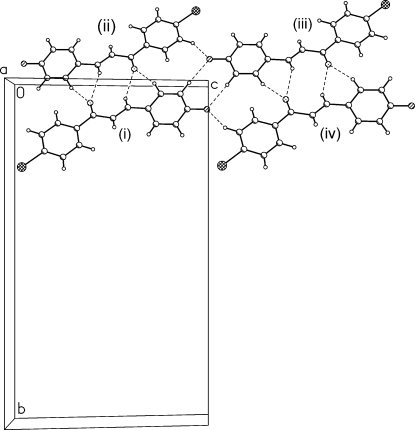
A fragment of the hydrogen bonded ribbon as seen approximately along the [100] direction. The hydrogen-bonding interactions are shown as dashed lines. Symmetry codes: (i) x, y, z; (ii) -x, -y, 1-z; (iii) 1-x, -y, 2-z; (iv) 1+x, y, 1+z.

**Fig. 3.**
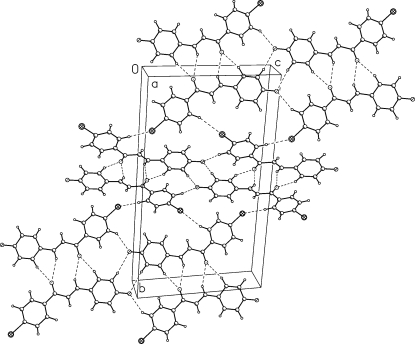
The crystal packing of the title compound showing neighbouring ribbons connected by weak C-H···Br contacts. Hydrogen-bonding interactions are shown as dashed lines.

## supplementary crystallographic information

### Comment

Chalcones or 1,3-diaryl-2-propen-1-ones (Ar—CH=CH—CO—Ar) are one of the major classes of natural products with widespread distribution in fruits, vegetables, spices, tea and soy based foodstuff. These compounds have been recently subjects of great interest for their interesting pharmacological activities (Dhar, 1981). They are finding application as organic nonlinear optical materials (NLO) for their SHG conversion efficiency (Sarojini *et al.*, 2006). Among several organic compounds reported which have NLO properties, chalcones derivatives are recognized material because of their excellent blue light transmittance and good crystallization ability (Goto *et al.*, 1991; Uchida *et al.* (1998); Indira *et al.*, 2002).

In the Cambridge Structural Database (Allen, 2002; Version 5.31, Nov. 2009) there are structural data for a series of the (2*E*)-1-(4-X)-3-(4-Y)prop-2-en-1-ones (X, Y - halogen). Those which have chloro- and/or bromo-substituents (X = Y = Cl: Wang *et al.*, 2005; X = Cl, Y = Br: Ng, Razak *et al.*, 2006; X = Y = Br: Ng, Shettigar *et al.*, 2006, and X = Br, Y = Cl: Yang *et al.*, 2006) are isostructural (P2_1_/c space group), and their crystal structure is similarly organized by π–π and halogen–halogen interactions. The only fluoro-derivative in the CCDC, (E)-1-(4-chlorophenyl)-3-(4-fluorophenyl)prop-2-en-1-one (i.e. X = Cl, Y = F, Qiu *et al.*, 2006) does not fit into this scheme; although it crystallizes in the same space group, however in different setting (P2_1_/n), both the molecular geometry and the crystal packing are different. Here we present the crystal structure of another fluoro-derivative, (2*E*)-1-(4-bromophenyl)-3-(4-fluorophenyl)prop-2-en-1-one (I, Scheme 1), which turned out to be isostructural with the latter example.

The crystal structure of I is isostructural with the F-Cl analogue; both compounds crystallize in the P2_1_/n space group and their crystal packings are almost identical. The isostructurality index (Kálmán *et al.*, 1991), which measures the differences in the positions of the atoms in the respective unit cell, is 0.9%, which is very close to the ideal value of 0.

The molecule of I is approximately planar (Fig. 1), the dihedral angle between the planes of the aromatic rings is 8.49 (13)°, and the central C=C-C=O fragment, is inclined by about 10° with respect to both rings. This conformation is of course similar to the F-Cl analogue, where the terminal planes make a angle of 9.1°, but is essentially different from the other group of halogeno- derivatives, where the conformation is much more folded, the dihedral angles being around 45°. It might be noted that there is also another difference: in the latter structures the angles between the central plane and the ring planes approximately sum up to the value of the angle between terminal planes. That means that in these cases the rings are twisted in opposite directions with respect to the central plane, while in I they are twisted in the same direction.

In the crystal structure, the molecules are connected by means of weak C—H···O hydrogen bonds into centrosymmetric dimers, and these dimers are connected by weak C—H···F interactions into two-molecule wide ribbons (Fig. 2). Neighbouring ribbons, inclined by an angle of approximately 58°, are connected by C—H···Br contacts (Fig. 3). In spite of the compounds without fluorine, there are no short halogen···halogen contacts. This might be regarded as another evidence of the essential difference between fluorine and other halogen atoms.

Additionally, thanks to the short unit cell parameter of 4.0060 (5)Å, the planes of molecules are stacked one onto another with an interplanar distance of about 3.53Å.

### Experimental

10 ml of 10% KOH was added to a mixture of 4-bromoacetophenone (0.01 mol) and p-fluorobenzaldehyde (0.01 mol) in 40 mL of ethyl alcohol. The reaction mixture was then kept under constant stirring. The solid product was filtered and recrystallized from an ethyl acetate solution at room temperature. C_15_H_10_BrFO, calculated: C 59.04% 3.30%, found: C 58.98, H 3.25. M.P.: 362-366 K.

### Refinement

The non-standard setting of space group P21/c (a=4.0060Å, b=23.125Å, c=14.4935Å, β=112.289°; transformation matrix 1 0 0 / 0 1 0 / -1 0 1) was chosen in order to be in accordance with the previously published isostructural structure. Hydrogen atoms were freely refined.

### Crystal data

**Table:**

C_15_H_10_BrFO	*F*(000) = 608
*M**_r_* = 305.14	*D*_x_ = 1.631 Mg m^−^^3^
Monoclinic, *P*2_1_/*n*	Cu *K*α radiation, λ = 1.54178 Å
Hall symbol: -P 2yn	Cell parameters from 4020 reflections
*a* = 4.0060 (5) Å	θ = 3.3–75.1°
*b* = 23.1253 (12) Å	µ = 4.49 mm^−^^1^
*c* = 13.4933 (9) Å	*T* = 295 K
β = 96.344 (6)°	Prism, colourless
*V* = 1242.36 (19) Å^3^	0.4 × 0.2 × 0.1 mm
*Z* = 4	

### Data collection

**Table:**

Oxford Diffraction SuperNova (single source at offset) Atlas diffractometer	2435 independent reflections
Radiation source: Nova (Cu) X-ray Source	2299 reflections with *I* > 2σ(*I*)
mirror	*R*_int_ = 0.018
Detector resolution: 5.2679 pixels mm^-1^	θ_max_ = 75.2°, θ_min_ = 3.8°
ω–scan	*h* = −3→5
Absorption correction: multi-scan (*CrysAlis PRO*; Oxford Diffraction, 2009)	*k* = −28→27
*T*_min_ = 0.386, *T*_max_ = 1.000	*l* = −16→14
4548 measured reflections	

### Refinement

**Table:**

Refinement on *F*^2^	Primary atom site location: structure-invariant direct methods
Least-squares matrix: full	Secondary atom site location: difference Fourier map
*R*[*F*^2^ > 2σ(*F*^2^)] = 0.043	Hydrogen site location: inferred from neighbouring sites
*wR*(*F*^2^) = 0.116	All H-atom parameters refined
*S* = 1.12	*w* = 1/[σ^2^(*F*_o_^2^) + (0.0695*P*)^2^ + 0.5227*P*] where *P* = (*F*_o_^2^ + 2*F*_c_^2^)/3
2435 reflections	(Δ/σ)_max_ < 0.001
203 parameters	Δρ_max_ = 0.73 e Å^−^^3^
0 restraints	Δρ_min_ = −0.50 e Å^−^^3^

### Special details

**Table:**

Geometry. All s.u.'s (except the s.u. in the dihedral angle between two l.s. planes) are estimated using the full covariance matrix. The cell s.u.'s are taken into account individually in the estimation of s.u.'s in distances, angles and torsion angles; correlations between s.u.'s in cell parameters are only used when they are defined by crystal symmetry. An approximate (isotropic) treatment of cell s.u.'s is used for estimating s.u.'s involving l.s. planes.
Refinement. Refinement of F^2^ against ALL reflections. The weighted R-factor wR and goodness of fit S are based on F^2^, conventional R-factors R are based on F, with F set to zero for negative F^2^. The threshold expression of F^2^ > 2σ(F^2^) is used only for calculating R-factors(gt) etc. and is not relevant to the choice of reflections for refinement. R-factors based on F^2^ are statistically about twice as large as those based on F, and R- factors based on ALL data will be even larger.

### Fractional atomic coordinates and isotropic or equivalent isotropic displacement parameters (Å^2^)

**Table:**

	*x*	*y*	*z*	*U*_iso_*/*U*_eq_	
Br1	0.64900 (8)	0.251119 (13)	0.06664 (2)	0.06378 (18)	
F1	0.8178 (6)	0.08073 (9)	0.99300 (12)	0.0843 (6)	
C1	0.7067 (7)	0.07919 (13)	0.89344 (19)	0.0548 (6)	
C2	0.5320 (8)	0.03177 (13)	0.85733 (18)	0.0544 (6)	
H2	0.507 (9)	−0.0022 (15)	0.902 (3)	0.073 (10)*	
C3	0.4195 (7)	0.03031 (11)	0.75622 (18)	0.0482 (6)	
H3	0.297 (9)	−0.0027 (13)	0.722 (3)	0.073 (10)*	
C4	0.4894 (6)	0.07548 (10)	0.69363 (16)	0.0400 (5)	
C5	0.6687 (7)	0.12313 (12)	0.73467 (19)	0.0489 (6)	
H5	0.725 (10)	0.1543 (15)	0.699 (3)	0.078 (10)*	
C6	0.7766 (8)	0.12519 (14)	0.8363 (2)	0.0572 (7)	
H6	0.914 (9)	0.1585 (15)	0.870 (3)	0.078 (11)*	
C7	0.3685 (6)	0.07087 (10)	0.58732 (16)	0.0417 (5)	
H7	0.190 (7)	0.0422 (13)	0.571 (2)	0.051 (8)*	
C8	0.4515 (6)	0.10426 (10)	0.51437 (17)	0.0423 (5)	
H8	0.627 (7)	0.1331 (13)	0.525 (2)	0.053 (8)*	
O9	0.1120 (5)	0.05436 (8)	0.38882 (13)	0.0589 (5)	
C9	0.3051 (6)	0.09403 (10)	0.41020 (17)	0.0401 (5)	
C10	0.3943 (5)	0.13412 (10)	0.32965 (16)	0.0368 (5)	
C11	0.2928 (6)	0.11865 (11)	0.23070 (17)	0.0426 (5)	
H11	0.184 (8)	0.0847 (13)	0.217 (2)	0.060 (8)*	
C12	0.3662 (6)	0.15324 (11)	0.15256 (17)	0.0462 (5)	
H12	0.294 (7)	0.1403 (12)	0.083 (2)	0.054 (8)*	
C13	0.5401 (6)	0.20384 (11)	0.17354 (17)	0.0427 (5)	
C14	0.6406 (6)	0.22126 (11)	0.27017 (19)	0.0461 (5)	
H14	0.716 (12)	0.2556 (13)	0.277 (3)	0.075 (13)*	
C15	0.5676 (6)	0.18557 (10)	0.34762 (17)	0.0436 (5)	
H15	0.646 (8)	0.1983 (13)	0.419 (2)	0.059 (8)*	

### Atomic displacement parameters (Å^2^)

**Table:**

	*U*^11^	*U*^22^	*U*^33^	*U*^12^	*U*^13^	*U*^23^
Br1	0.0534 (3)	0.0764 (3)	0.0619 (3)	0.00176 (12)	0.00788 (16)	0.03331 (14)
F1	0.1142 (16)	0.1010 (15)	0.0343 (8)	−0.0239 (12)	−0.0074 (9)	−0.0034 (8)
C1	0.0614 (16)	0.0684 (16)	0.0341 (12)	−0.0027 (13)	0.0027 (10)	−0.0040 (11)
C2	0.0702 (17)	0.0569 (15)	0.0362 (12)	−0.0062 (13)	0.0067 (11)	0.0058 (11)
C3	0.0589 (14)	0.0470 (13)	0.0386 (11)	−0.0078 (11)	0.0049 (10)	0.0009 (10)
C4	0.0422 (12)	0.0436 (11)	0.0344 (10)	0.0012 (9)	0.0052 (8)	0.0013 (9)
C5	0.0573 (15)	0.0465 (13)	0.0429 (12)	−0.0077 (11)	0.0059 (10)	0.0020 (10)
C6	0.0648 (17)	0.0624 (16)	0.0437 (13)	−0.0148 (13)	0.0031 (12)	−0.0074 (12)
C7	0.0449 (12)	0.0435 (11)	0.0365 (11)	−0.0004 (9)	0.0036 (9)	−0.0011 (9)
C8	0.0441 (12)	0.0455 (12)	0.0368 (11)	−0.0024 (10)	0.0021 (9)	−0.0005 (9)
O9	0.0744 (13)	0.0577 (11)	0.0428 (9)	−0.0259 (10)	−0.0017 (8)	0.0021 (8)
C9	0.0440 (12)	0.0411 (11)	0.0351 (10)	−0.0001 (9)	0.0038 (8)	−0.0003 (9)
C10	0.0357 (11)	0.0393 (11)	0.0349 (10)	0.0039 (8)	0.0019 (8)	−0.0005 (8)
C11	0.0483 (13)	0.0418 (12)	0.0366 (11)	−0.0031 (9)	0.0000 (9)	−0.0012 (9)
C12	0.0499 (13)	0.0536 (13)	0.0337 (11)	0.0025 (10)	−0.0014 (9)	0.0032 (9)
C13	0.0360 (11)	0.0487 (12)	0.0432 (11)	0.0063 (9)	0.0037 (8)	0.0111 (9)
C14	0.0435 (12)	0.0430 (13)	0.0513 (13)	−0.0063 (10)	0.0027 (10)	0.0005 (10)
C15	0.0474 (12)	0.0454 (12)	0.0372 (11)	−0.0032 (10)	0.0008 (9)	−0.0032 (9)

### Geometric parameters (Å, °)

**Table:**

Br1—C13	1.899 (2)	C8—C9	1.481 (3)
F1—C1	1.368 (3)	C8—H8	0.97 (3)
C1—C6	1.361 (4)	O9—C9	1.214 (3)
C1—C2	1.362 (4)	C9—C10	1.501 (3)
C2—C3	1.389 (3)	C10—C15	1.385 (3)
C2—H2	1.00 (3)	C10—C11	1.399 (3)
C3—C4	1.391 (3)	C11—C12	1.381 (3)
C3—H3	0.99 (3)	C11—H11	0.91 (3)
C4—C5	1.396 (3)	C12—C13	1.375 (4)
C4—C7	1.466 (3)	C12—H12	1.00 (3)
C5—C6	1.392 (4)	C13—C14	1.381 (3)
C5—H5	0.91 (4)	C14—C15	1.388 (3)
C6—H6	1.02 (4)	C14—H14	0.85 (3)
C7—C8	1.322 (3)	C15—H15	1.02 (3)
C7—H7	0.98 (3)		
			
C6—C1—C2	123.8 (2)	C9—C8—H8	117.1 (18)
C6—C1—F1	118.1 (3)	O9—C9—C8	121.4 (2)
C2—C1—F1	118.1 (3)	O9—C9—C10	119.4 (2)
C1—C2—C3	118.0 (2)	C8—C9—C10	119.2 (2)
C1—C2—H2	119.8 (19)	C15—C10—C11	118.3 (2)
C3—C2—H2	122.0 (19)	C15—C10—C9	123.94 (19)
C2—C3—C4	120.9 (2)	C11—C10—C9	117.8 (2)
C2—C3—H3	124 (2)	C12—C11—C10	121.1 (2)
C4—C3—H3	115 (2)	C12—C11—H11	119 (2)
C3—C4—C5	118.8 (2)	C10—C11—H11	120 (2)
C3—C4—C7	118.2 (2)	C13—C12—C11	118.8 (2)
C5—C4—C7	123.0 (2)	C13—C12—H12	122.5 (17)
C6—C5—C4	120.5 (2)	C11—C12—H12	118.7 (17)
C6—C5—H5	115 (2)	C12—C13—C14	122.0 (2)
C4—C5—H5	124 (2)	C12—C13—Br1	119.16 (18)
C1—C6—C5	118.1 (3)	C14—C13—Br1	118.80 (19)
C1—C6—H6	118 (2)	C13—C14—C15	118.3 (2)
C5—C6—H6	124 (2)	C13—C14—H14	116 (3)
C8—C7—C4	127.1 (2)	C15—C14—H14	125 (3)
C8—C7—H7	118.0 (16)	C10—C15—C14	121.5 (2)
C4—C7—H7	114.6 (16)	C10—C15—H15	120.5 (17)
C7—C8—C9	120.5 (2)	C14—C15—H15	118.0 (17)
C7—C8—H8	122.0 (18)		
			
C6—C1—C2—C3	0.1 (5)	O9—C9—C10—C15	169.3 (2)
F1—C1—C2—C3	−180.0 (3)	C8—C9—C10—C15	−9.8 (3)
C1—C2—C3—C4	1.2 (4)	O9—C9—C10—C11	−10.0 (3)
C2—C3—C4—C5	−1.5 (4)	C8—C9—C10—C11	170.8 (2)
C2—C3—C4—C7	179.0 (2)	C15—C10—C11—C12	0.7 (4)
C3—C4—C5—C6	0.3 (4)	C9—C10—C11—C12	−179.8 (2)
C7—C4—C5—C6	179.9 (3)	C10—C11—C12—C13	−0.4 (4)
C2—C1—C6—C5	−1.3 (5)	C11—C12—C13—C14	−0.6 (4)
F1—C1—C6—C5	178.9 (3)	C11—C12—C13—Br1	178.96 (18)
C4—C5—C6—C1	1.0 (5)	C12—C13—C14—C15	1.3 (4)
C3—C4—C7—C8	−168.8 (3)	Br1—C13—C14—C15	−178.27 (18)
C5—C4—C7—C8	11.6 (4)	C11—C10—C15—C14	0.0 (4)
C4—C7—C8—C9	−179.7 (2)	C9—C10—C15—C14	−179.4 (2)
C7—C8—C9—O9	−1.5 (4)	C13—C14—C15—C10	−1.0 (4)
C7—C8—C9—C10	177.7 (2)		

### Hydrogen-bond geometry (Å, °)

**Table:**

*D*—H···*A*	*D*—H	H···*A*	*D*···*A*	*D*—H···*A*
C7—H7···O9^i^	0.98 (3)	2.62 (3)	3.512 (3)	151 (2)
C3—H3···O9^i^	0.99 (3)	2.41 (3)	3.358 (3)	160 (2)
C15—H15···Br1^ii^	1.02 (3)	2.92 (3)	3.845 (2)	151 (2)
C12—H12···F1^iii^	1.00 (3)	2.55 (3)	3.351 (3)	137 (2)

Symmetry codes: (i) −*x*, −*y*, −*z*+1; (ii) *x*+1/2, −*y*+1/2, *z*+1/2; (iii) *x*−1, *y*, *z*−1.

## References

[bb1] Allen, F. H. (2002). *Acta Cryst.* B**58**, 380–388. 10.1107/s0108768102003890 12037359

[bb2] Altomare, A., Cascarano, G., Giacovazzo, C. & Guagliardi, A. (1993). *J. Appl. Cryst* **26**, 343–350.

[bb3] Dhar, D. N. (1981). *The Chemistry of Chalcones and Related Compounds* New York: John Wiley.

[bb4] Goto, Y., Hayashi, A., Kimura, Y. & Nakayama, M. (1991). *J. Cryst. Growth*, **108**, 688–698.

[bb5] Indira, J., Karat, P. P. & Sarojini, B. K. (2002). *J. Cryst. Growth*, **242**, 209–214.

[bb6] Kálmán, A., Argay, G., Scharfenberg-Pfeiffer, D., Höhne, E. & Ribár, B. (1991). *Acta Cryst.* B**47**, 68–77.

[bb7] Ng, S.-L., Razak, I. A., Fun, H.-K., Shettigar, V., Patil, P. S. & Dharmaprakash, S. M. (2006). *Acta Cryst.* E**62**, o2175–o2177.

[bb8] Ng, S.-L., Shettigar, V., Razak, I. A., Fun, H.-K., Patil, P. S. & Dharmaprakash, S. M. (2006). *Acta Cryst.* E**62**, o1421–o1423.

[bb9] Oxford Diffraction (2009). *CrysAlis PRO* Oxford Diffraction Ltd, Yarnton, England.

[bb10] Qiu, X.-Y., Luo, Z.-G., Yang, S.-L. & Liu, W.-S. (2006). *Acta Cryst.* E**62**, o3525–o3526.

[bb11] Sarojini, B. K., Narayana, B., Ashalatha, B. V., Indira, J. & Lobo, K. G. (2006). *J. Cryst. Growth*, **295**, 54–59.

[bb12] Sheldrick, G. M. (2008). *Acta Cryst.* A**64**, 112–122. 10.1107/S0108767307043930 18156677

[bb13] Siemens (1989). *XP* Siemens Analytical X-ray Instruments Inc., Madison, Wisconsin, USA.

[bb14] Uchida, T., Kozawa, K., Sakai, T., Aoki, M., Yoguchi, H., Abduryim, A. & Watanabe, Y. (1998). *Mol. Cryst. Liq. Cryst* **315**, 135–140.

[bb15] Wang, L., Yang, W. & Zhang, D.-C. (2005). *Acta Cryst.* E**61**, o2820–o2822.

[bb16] Yang, W., Wang, L. & Zhang, D. (2006). *J. Chem. Crystallogr.* **36**, 195–198.

